# Wafer-scale CMOS foundry silicon-on-insulator devices for integrated temporal pulse compression

**DOI:** 10.1515/nanoph-2025-0481

**Published:** 2025-11-05

**Authors:** Ju Won Choi, Kenny Y.K. Ong, Masaki Kato, Gemma Y.N. Chee, Benjamin J. Eggleton, Radhakrishnan Nagarajan, Dawn T.H. Tan

**Affiliations:** 233793 Photonics Devices and Systems Group, Singapore University of Technology and Design, 8 Somapah Rd., Singapore 487372, Singapore; Marvell Asia Pte. Ltd., Tai Seng Centre #10-01, 3 Irving Road, Singapore 369522, Singapore; Methodist Girls’ School, 11 Blackmore Dr, Singapore 599986, Singapore; Institute of Photonics and Optical Science, School of Physics, The University of Sydney, Camperdown, NSW 2006, Australia; The University of Sydney Nano Institute (Sydney Nano), The University of Sydney, Camperdown, NSW 2006, Australia

**Keywords:** silicon photonics, nonlinear optics, temporal pulse compression, wafer-scale manufacturing

## Abstract

Optical pulses are essential as information carriers, for driving nonlinear light sources, imaging, the study of attosecond science and 3D printing. In many applications, short pulses are needed. For example, the resolution of imaging methods which utilize short pulses is limited by the temporal width of the pulses, as is the capacity of time division multiplexed data. The temporal compression of optical pulses is an important approach to achieving ultrashort pulses. With the widespread proliferation of silicon photonics and their use in a multitude of applications, an integrated, CMOS-compatible approach for pulse compression would allow its seamless integration with other photonic integrated circuits. In this work, we experimentally demonstrate silicon-based pulse compression fabricated in a CMOS foundry. The first technique utilizes two stages, one for generating self-phase modulation through the Kerr nonlinearity in silicon, and the second for temporal synchronization of the new wavelengths. The second technique leverages Bragg soliton-effect temporal compression. We experimentally demonstrate temporal compression of up to 3.6× and good agreement with numerical calculations. This work represents efficient silicon-on-insulator devices for temporal compression realized using a wafer-scale CMOS foundry process and may therefore be mass manufactured and integrated seamlessly with other photonic and electronic circuits.

## 1 Introduction

Temporal compression of optical pulses may leverage the Kerr effect, facilitating access to ultrashort pulses required for a variety of applications including optical imaging and high-speed communications based on optical time division multiplexing (OTDM) [1], [2], [3], [4], [5], [6]. OTDM may further be combined with wavelength multiplexing to augment the aggregate data rate [6], while the temporal compression of optical pulses allows a larger number of pulse trains to be interleaved to achieve a higher overall OTDM rate [7]. Earliest demonstrations of temporal compression utilized bulk optics. In 1969, Treacy reported compression using a diffraction grating pair [8]. Temporal compression involving the Kerr effect was reported by Fischer et al. that same year, forming the foundations for a compression method which is widely used today [9]. In that work, temporal compression utilized nonlinear phase acquisition in an optical Kerr liquid followed by differential optical delays. With advancements in the manufacture of low-loss, high-quality single mode fiber, methods for temporal compression which were once limited to theory could be experimentally demonstrated. Theoretical predictions of fiber-based self-phase modulation combined with a grating pair could be used to temporally compress pulses [10]. In this method of optical pulse compression, the optical pulse undergoes a frequency chirp, prior to rephasing with a dispersive element. This approach decouples nonlinearity and dispersion. In integrated photonics, a waveguide is analogous to bulk crystal used for nonlinear phase acquisition whereas a chirped on-chip grating is used in place of a bulk grating.

In parallel, seminal work from Molleneur set the stage for decades of advancements in optical soliton science, providing new approaches for short pulse generation [11]. Today, the field of solitons is flourishing not just within the optical fiber community but also in integrated photonics. Short pulses may be generated through soliton effects via the initial temporal narrowing experienced by a high order soliton. Contrary to compression using self-phase modulation prior to propagation through a dispersive element, in soliton-effect compression, the nonlinearity and dispersion are distributed over the fiber or waveguide. Bragg-soliton effect compression facilitates strong compression dynamics through the strong grating induced dispersion on the stopband edge [12], [13]. In particular, Bragg soliton phenomena in chip-scale platforms have undergone tremendous advancements in the last few years due to improvements in nanofabrication and the availability of high nonlinear figure of merit platforms [14], [15], [16], [17]. Compared to optical fiber, chip-scale devices may have nonlinear parameters six orders of magnitude larger, enabling significantly lower power operation and smaller form factors to be used [14], [18].

Significant progress has been made in both types of compression in chip-scale devices [19], [20], [21], [22], [23], [24]. In many of these demonstrations, devices with sub-100 nm critical dimensions were required. To achieve the requisite fabrication resolution, electron-beam (e-beam) lithography is typically used. While e-beam allows very high-resolution patterning to be performed, it requires line by line writing which limits the throughput. Conversely, the use of processing techniques which adopt optical lithography (e.g. UV lithography) such as that used in CMOS foundries allows very high throughput manufacture of photonic devices. The research question which needs to be answered is whether designs which work within the limited resolutions available in wafer-scale processes may be successfully established and reduced to practice.

In this paper, we explore nonlinear temporal pulse compression fabricated using an 8-inch wafer-scale CMOS foundry process. We present the design and experimental characterization of two types of nonlinear phenomena for temporal compression in integrated devices, achieving a compression factor of up to 3.6×. We further explore how foundry limitations may be overcome to achieve the required device performance and device limitations arising from the material platform. These results showcase the widescale manufacturability of such devices which thus far have been mainly limited to realization using e-beam lithography and may accelerate their implementation in more complex photonic integrated circuits for applications in high-speed communications and optical sources.

## 2 Device design

**Figure 1:**
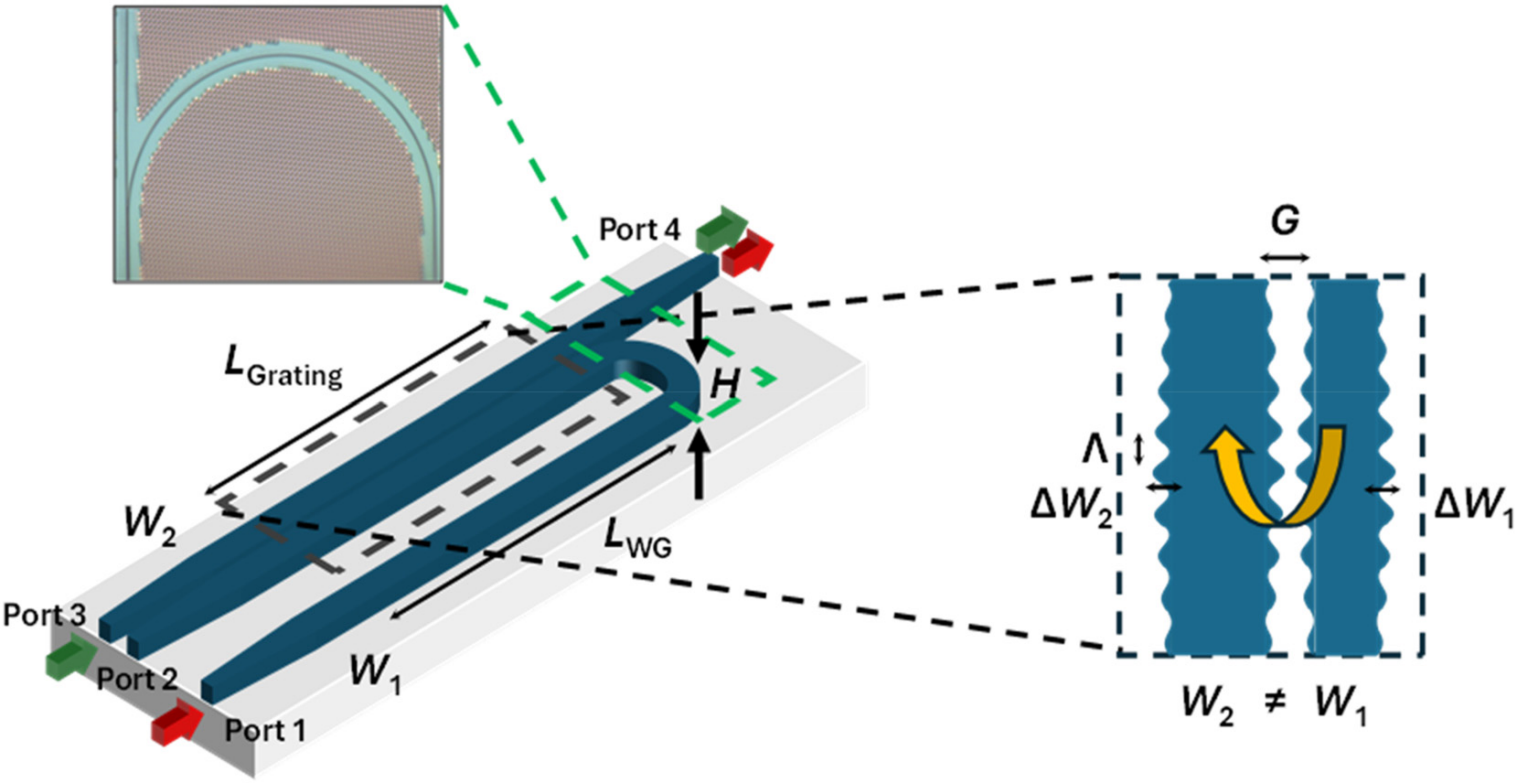
Schematic of the device used for temporal compression. An optical micrograph of the device is shown. Configuration 1 refers to an input at Port 3 and output at Port 4. Configuration 2 refers to an input at Port 1 and output at Port 4.

Figure 1 shows the schematic of the device studied in this paper. The device is implemented on the silicon-on-insulator platform with a height, *H* = 220 nm. All devices have SiO_2_ under- and over-cladding. Though this is a prolific material in CMOS-wafer scale fabrication of photonics devices, silicon has some drawbacks in nonlinear efficiency due to its small bandgap of 1.1 eV. This results in two-photon absorption (TPA) and free-carrier absorption (FCA) at wavelengths shorter than 2.2 μm including the important telecommunications wavelengths [25], [26]. We analyze the impact of these effects through numerical simulations later. The device shown in Figure 1(a) consists of two sinusoidally modulated gratings which are coupled. The coupled grating has widths, *W*_1_ = 400 nm and *W*_2_ = 500 nm and a length of 4 mm. The maximum modulation amplitude for *W*_1_ and *W*_2_ is 40 nm and 50 nm respectively. The gap between the modulation peaks is 150 nm which is selected such that it may be well resolved by the 8-inch CMOS foundry process used to fabricate the devices. The coupled grating’s modulation amplitude varies from its maximum value at the center of the grating to 0 at the ends in a raised cosine profile to achieve apodization. The average grating pitch, Λ_0_ is selected to have a stopband within the C-band and varies linearly along the length of the coupled grating according to the expression, $\Lambda \left(z\right)=\Lambda_{0}+\Delta \Lambda \cdot \frac{z}{L}$ . Figure 1(a) further designates Ports 1–4.

**Figure 2:**
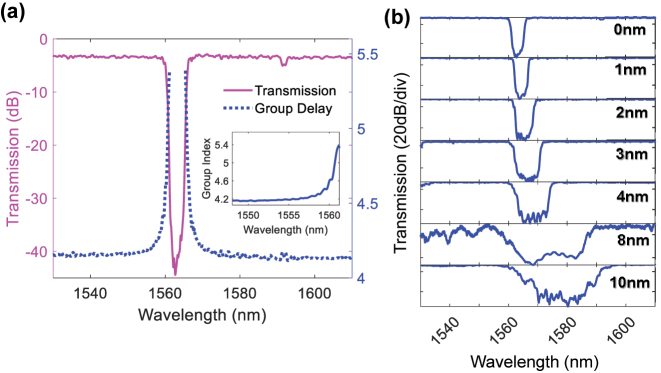
Optical properties of the device when using configuration 1. (a) Measured transmission spectrum and group delay profile for the silicon grating used for temporal compression. The inset shows the group index as a function of wavelength. (b) Measured transmission spectrum of silicon gratings with for ΔΛ from 0 nm to 10 nm. It may be observed that the bandwidth of the stopband increases with ΔΛ.

We describe the two configurations for using the device for temporal compression. Configuration 1 involves launching input pulses at Port 3 and pulses undergo Bragg-soliton effect compression prior to exiting at Port 4. In this configuration, the single grating with width, *W*_2_ generates a stopband within the C-band. The stopband is centered at wavelength, ***λ***_***B***,1_, is governed by the equation, ***λ***_***B***,1_ = 2 × ***n***_**eff**,2_ × **Λ**, where *n*_eff2_ is the effective index of the grating with width *W*_2_. The blue-side of the grating stopband is characterized as having a rapidly increasing group index as wavelength is increased. Figure 2(a) shows the measured transmission and group index spectrum for the grating with ΔΛ = 0. The transmission spectrum is measured using a broadband light source adjusted for transverse electric polarization and an optical spectrum analyzer, whereas the group index spectrum is measured using a dispersion analyzer. It may be observed that as the wavelength increases from 1556 nm to 1561 nm, the group index undergoes a large increase from 4.2 to 5.4, further indicating a large anomalous dispersion. This large dispersion originates from the interaction between the forward and backward propagating grating modes that are a result of the grating periodicity [27]. In addition, the elevated group index results in an augmented effective nonlinear parameter, $\gamma_{\text{eff}}=\gamma {\left(\frac{n_{g}}{n_{0}}\right)}^{2}$ , where *γ*, *n*_g_ and *n*_0_ refer to the nonlinear parameter, group index and core refractive index, respectively. The strong nonlinearity and anomalous dispersion distributed along the grating length leads to Bragg soliton-effect compression when the pulse wavelength is located on the blue side of the grating stopband. Since the apodization is implemented by gradually increasing the modulation amplitude from zero at its ends to its maximum value of 50 nm in the center in a raised cosine profile, it is not clear if the resolution available in the wafer-scale process is sufficient to implement it effectively. It may be observed that there is minimal ripple in the transmission spectrum owing to the apodization being effectively implemented. Figure 2(b) further shows the measured transmission spectra for ΔΛ from 0 nm to 10 nm. It may be observed that the bandwidth of the stopband increases with ΔΛ.

The second configuration for temporal compression (configuration 2), involves launching input pulses into Port 1 and an output at Port 4. In this configuration, the wavelength of the input pulses needs to be located within the grating stopband. The optical pulses will first undergo self-phase modulation in the 4 mm long silicon waveguide with width *W*_1_. The spectrally broadened pulses will next enter the coupled grating, where cross coupling between gratings with width *W*_1_ and *W*_2_ will generate a stopband centered at wavelength, $\lambda_{B,2}=\left(n_{e\mathrm{ff},1}+n_{e\mathrm{ff},2}\right)\cdot \Lambda$ , where *n*_eff,1_ refers to the effective index of the grating with width *W*_1_. Light at wavelengths within the coupled grating’s stopband will couple to the adjacent grating (*W*_2_) along the coupled grating’s length. Any uncoupled light will exit at Port 2. When ΔΛ has a positive, non-zero value, anomalous dispersion will be generated. The distance over which the different wavelengths of light will travel will differ and give rise to a linear differential group delay. The spectrally broadened pulses will undergo temporal rephasing such that the blue (red) components will be accelerated (delayed) and approach the center of the pulse. This form of temporal compression decouples the nonlinear and dispersive effects and is a distinct compression mechanism from configuration 1. We note further that the stopband locations for configurations 1 and 2 of each device are different by virtue of the different governing Bragg conditions.

We design and fabricate several devices with different values of ΔΛ. All gratings have a length of 4 mm. The transmission spectrum of the gratings when using configuration 1 (input at Port 3 and output at Port 4) is first measured using an amplified spontaneous emission source and optical spectrum analyzer. Light from the source is first aligned for transverse-electric (TE) polarization. Figure 2(b) shows the measured transmission spectra of the gratings where minimal ripple exists in the transmission spectrum. It may also be observed that the bandwidth of grating stopband increases with ΔΛ. The transmission and group delay spectra of the grating with ΔΛ = 0 nm used for the temporal compression measurements in configuration 1 are shown in Figure 2(a). The group delay spectrum is measured using a dispersion analyzer.

**Figure 3:**
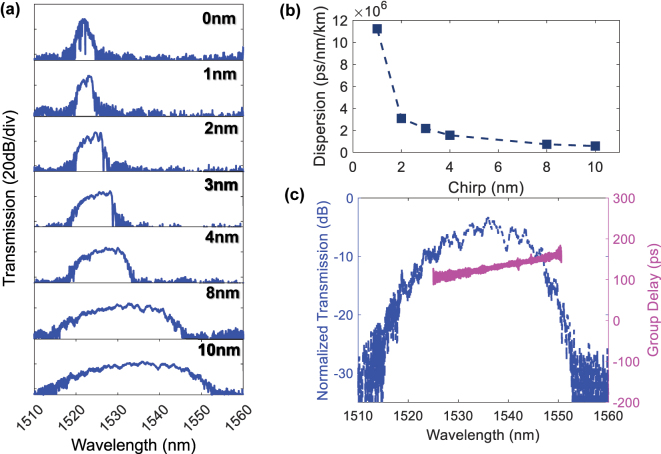
Optical properties of the device when using configuration 2. (a) The measured transmission spectrum and (b) dispersion for Configuration 2 as a function of chirp. (c) The measured transmission and group delay spectrum within the grating stopband for ΔΛ = 10 nm.

Next, the measured transmission spectra for configuration 2 for the devices with different values of ΔΛ are shown in Figure 3(a). It may be observed that the bandwidth of the stopband increases with ΔΛ. The measured dispersion within the stopband as a function of ΔΛ is further shown in Figure 3(b). The achievable dispersion within the range of devices 5.7 × 10^5^ ps/nm/km to 1.1 × 10^7^ ps/nm/km. Figure 3(c) shows the measured transmission and group delay spectrum for ΔΛ = 10 nm, corresponding to a dispersion of 5.7 × 10^5^ ps/nm/km, which will be used for demonstrating pulse compression. It may be observed that the group delay within the stopband is linear, with the delay increasing with wavelength, generating anomalous dispersion.

## 3 Temporal compression characterization

**Figure 4:**
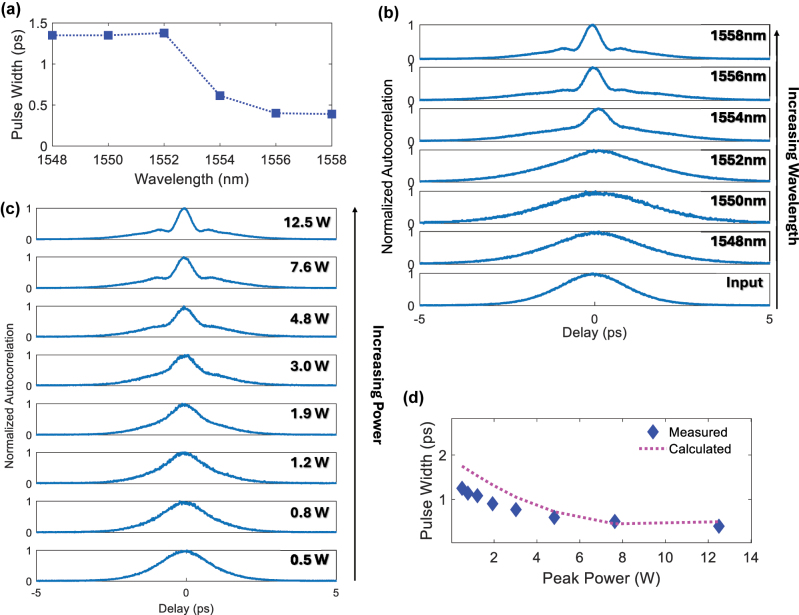
Experimentally measured wavelength dependent temporal compression for configuration 1. (a) The output pulse width and (b) the normalized autocorrelation as a function of input pulse wavelength. (c) Experimentally measured autocorrelation traces. (d) Measured (blue diamonds) and calculated (fuchsia dashed line) output pulse width as a function of input peak power for 1.4 ps pulses centered at 1558 nm.

We first study configuration 1 for temporal compression of pulses using the grating shown in Figure 2(a). In this configuration, Bragg soliton-effect compression is leveraged. The wavelength of the optical pulses is located on the blue side of the stopband edge, varied between 1548 nm–1558 nm. We first characterize the output of 1.4 ps pulses at a fixed input peak power of 12.5 W as a function of the pulse wavelength. The measured output pulse width is shown in Figure 4(a) while Figure 4(b) shows the autocorrelation traces as a function of the wavelength of the input pulses. It may be observed that the compression strengthens as the pulse wavelength approaches the stopband centered at 1565 nm. The strongest compression is observed at a wavelength of 1558 nm where the pulses are compressed to a pulse width of 0.39 ps, corresponding to a compression factor, $CF=\frac{Input\;Pulse\;FWHM}{Output\;Pulse\;FWHM}$ of 3.6×. At this wavelength, the group delay changes most rapidly with wavelength, indicating the strongest anomalous dispersion and largest group index. We perform further characterization with an input pulse wavelength of 1558 nm as a function of input pulse peak power. In Bragg soliton-effect compression, higher input peak powers result in larger soliton orders, $N=\sqrt{\left(\frac{T_{0}^{2}P_{0}\gamma_{\text{eff}}}{\left|\beta_{2}\right|}\right)}$ , where $T_{0}\approx \frac{T_{\text{FWHM}}}{1.665}$ for hyperbolic secant pulses and *T*_FWHM_ is the pulse width [28]. At higher soliton orders, the optical pulses experience a stronger initial temporal narrowing prior to splitting and recombining within a soliton period. Figure 4(c) and (d) show the temporal compression strengthening with increasing power.

**Figure 5:**
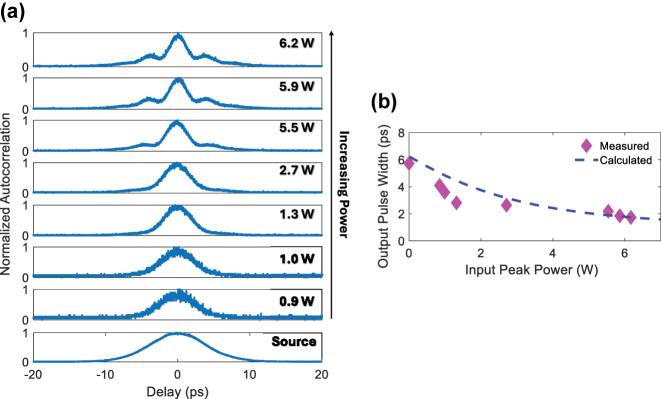
Experimentally measured temporal compression for configuration 2. (a) The measured autocorrelation traces as a function of input peak power of 5.7 ps input pulses for Configuration 2, from lowest (bottom) to highest (top) input peak power. (b) The experimentally measured (fuchsia diamonds) and numerically calculated (blue dashed line) compressed pulse width as a function of input peak.

Next, we study configuration 2 using the device shown in Figure 3(c) for temporal compression. In these experiments, 5.7 ps pulses are coupled into Port 1 and the output measured at Port 4. The pulses are centered at a wavelength of 1544 nm so as to be located well within the stopband of the grating where transmission is high and the group delay profile is linear. The output pulses are measured with an autocorrelator as a function of the input peak power of the pulses. Figure 5(a) shows the autocorrelation traces as the input peak power of the pulses is increased. The output pulse width as a function of power is shown in Figure 5(b), where it may be seen that the pulses are compressed to 1.7 ps at an input peak power of 6.2 W, representing 3.4× compression of 5.7 ps pulses.

Next, we perform numerical calculations using the generalized nonlinear Schrödinger equation (GNLSE). The GNLSE may be described by the following expression [28], [29]:

(1) $$\begin{array}{ll} \frac{\partial A}{\partial z} & =-\frac{\alpha }{2}A+i\overset{3}{\underset{k=2}{\sum }}\beta_{k}\frac{\partial^{k}A}{\partial T^{k}}=i\gamma_{e\mathrm{ff}}\left[{\left|A\right|}^{2}A\right]\\ & \;-\frac{\beta_{TPA}}{2}\left[{\left|A\right|}^{2}A\right]+\left[ik_{c}-\frac{\sigma }{2}\right]N\left(z,t\right)A \end{array}$$

Here, we assume a slowly varying pulse envelope, *A*(*z*, *t*). *α*, *β* and *γ*_eff_ refer to the experimentally measured loss coefficient, dispersion and effective nonlinear parameter, respectively. The effective nonlinear parameter is derived from the experimentally measured group index and reported *n*_2_ for silicon [25], [30]. *β*_TPA_, *σ* and *k*_c_ denote the TPA coefficient, FCA cross section and the free-carrier dispersion coefficient, respectively [29]. The free carrier density, *N*(*z*,*t*) varies as a function of the propagation distance and time, described by the expression, $\frac{\partial N}{\partial t}=\frac{\beta_{TPA}}{2\hbar \omega A_{\text{eff}}^{2}}{\left|A\right|}^{4}-\frac{N}{\tau_{c}}$ , where ℏ is Planck’s constant, *ω* is the angular frequency and *A*_eff_ is the effective area of the mode.

We first calculate the output pulse widths as a function of input power for configuration 1 using the experimental conditions used for Figure 4(c) and (d). The calculated values are plotted on Figure 4(d) as the dotted fuchsia line. Good agreement with the measured values is obtained, corroborating the observed trend of decreasing pulse width with an increase in the pulse peak power.

The output pulse widths for configuration 2 as a function of input peak power corresponding to the experimental conditions used for Figure 5(a) and (b) are calculated and plotted in Figure 5(b) as the blue dashed line. Good agreement with the measured data is achieved.

## 4 Discussion

In this work, an 8-inch CMOS foundry process was leveraged for fabrication of the devices. This work showcases the feasibility of wafer-scale manufacturing to realize devices which have in the past involved designs with very small critical dimensions, requiring low throughput lithographic approaches such as electron-beam lithography. However, some inherent trade-offs and limitations associated with the wafer-scale process exist which needed to be accounted for in the device design.

(1) Due to resolution limitations, the coupled grating was designed to have a gap of 150 nm, which was around the smallest value that could be resolved. For strong cross-coupling, the condition, *κ.L* >> π where *κ* is the cross-coupling coefficient needs to be satisfied to ensure that most of the light undergoes cross coupling [31]. If this condition is not satisfied, the optical loss incurred through incomplete cross-coupling will be large. In previous reports of coupled gratings fabricated using electron-beam lithography, gaps of tens of nanometers could be used and the condition could be easily satisfied. In this work, due to limitations to the critical dimensions, the grating length needed to be extended to ensure that *κ.L* >> π is satisfied. In addition, as a result of the longer grating length required, the resulting dispersion could have a large magnitude that would exceed that required for optimal compression. A trade-off with the selected value of ΔΛ would help to counter this effect. In addition, we note that with the small gap of 150 nm, non-conformal deposition of the oxide cladding in the deposition could occur which may lead to some air gaps. The air gaps may cause a slight modification in the coupling between the coupled gratings. As the wafer-scale process is well controlled and consistent from across different runs, this artifact can be accounted for in the design and not pose issues for reliability.
(2) The resolution limitation of the wafer-scale process also restricted the type of grating modulation that could be used. Previous reports of gratings used for Bragg soliton effects utilized a periodic effective index modulation realized with pillars placed a distance, *G*(*z*) away from a central waveguide with the smallest value of *G* being 50 nm [17]. Since this value of *G* is too small to be resolved by the 8-inch process, the grating designs in this work utilized sinusoidal sidewall modulation which did not require small gaps. The gap of 150 nm between the coupled gratings could be resolved, enabling the good performance of the temporal compression device. In addition, one question which needed to be answered was whether the apodization which required the modulation amplitude to increase from a value of 0 nm at the ends to 50 nm in the center could be well resolved. The smooth transmission spectrum achieved for the gratings (Figure 2(b)) with minimal out of band ripple showcases the effectiveness of the apodization.
(3) Of particular significance in this work is the use of silicon for implementation of the wafer-scale fabricated devices. In wafer-scale processing of photonic integrated circuits, silicon and silicon nitride are two common materials that are used for waveguide devices. Of the two, silicon waveguides have a significantly larger nonlinear parameter, 100× larger than silicon nitride waveguides and implementing the device on silicon allows lower powers and shorter device footprints. However, it is well known that silicon suffers from TPA and FCA at telecommunications wavelengths. The efficiency of nonlinear phase acquisition is sub-optimal compared to other platforms such as ultra-silicon-rich nitride which may be grown using low-temperature, CMOS-compatible processes but are not widely available yet in foundries [18], [32].

**Figure 6:**
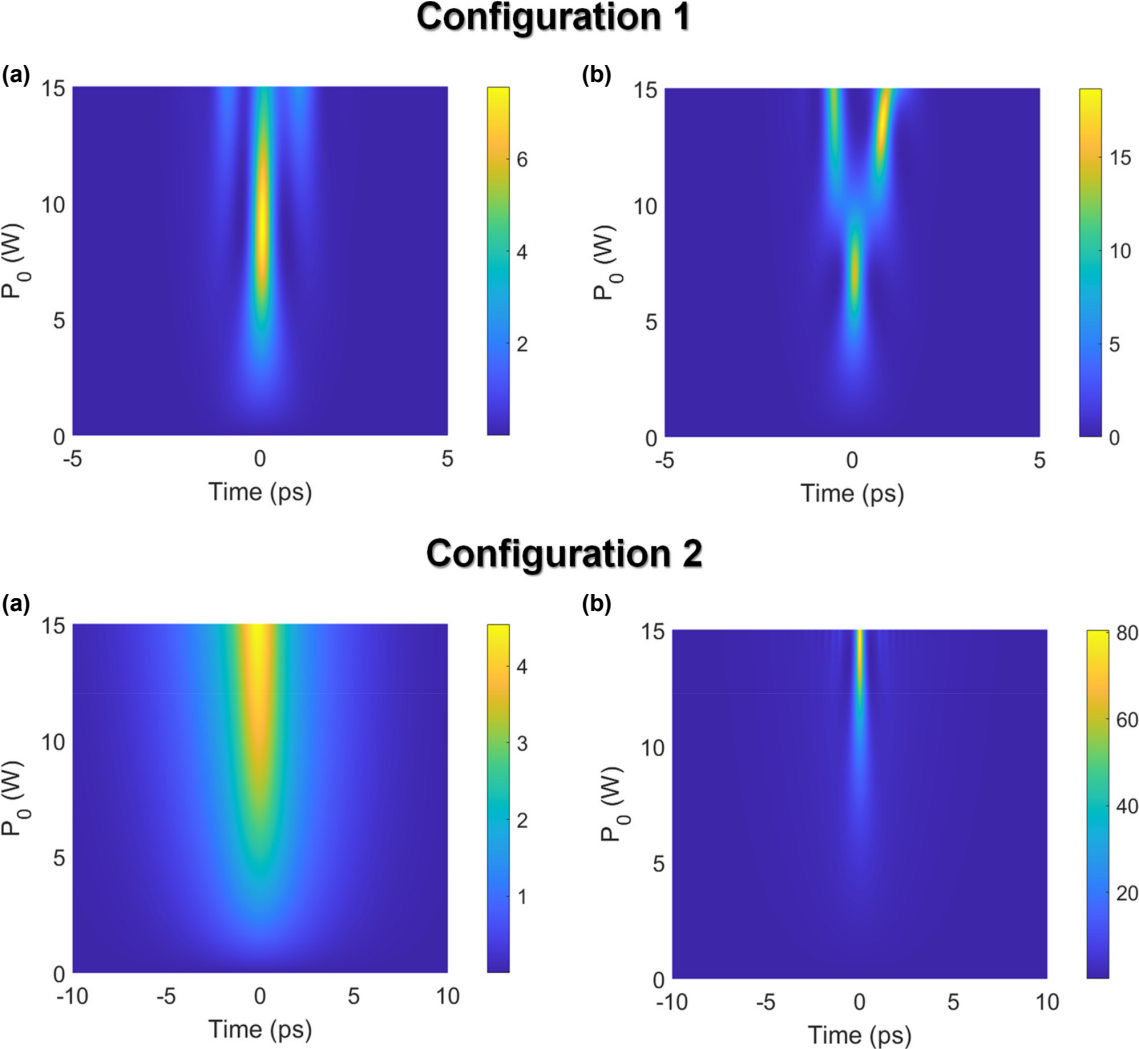
Numerical calculations of configurations 1 and 2. The output pulse profile as a function of input pulse peak power for configuration 1 using the input pulse width of 1.4 ps used in the experiments, in the (a) presence and (b) absence of TPA and FCA. The output pulse profile as a function of input pulse peak power for configuration 2 using the input pulse width of 5 ps used in the experiments, in the (a) presence and (b) absence of TPA and FCA.

To study the impact of TPA and FCA, we utilize Eq. (1) to observe the output pulse profile as a function if input peak power for Configurations 1 and 2 in the presence and absence of TPA and FCA. Figure 6(a) and (b) simulate the temporal pulse profile as the input peak power is varied between 0.001 W (low power) and 15 W for Configuration 1, using the conditions used for experiments in

Figures 4 and 6(a) simulates the pulse profile in the presence of TPA and FCA. It may be seen that for an input peak power range between 0.001 W and 15 W, temporal compression occurs. However, the highest peak power of the output pulse is 7.5 W, corresponding to an input peak power of 9 W. This suggests that while temporal compression has successfully reduced the pulse width, the attenuation in the pulse power has led to the peak power of the compressed pulse being even lower than the uncompressed pulse.

Conversely, Figure 6(b) simulates the pulse profile as a function of input peak power in the absence of TPA and FCA. The output pulse width is observed to monotonically decrease up to an input peak power of 7.2 W. The shortest pulse width and the peak power at which it occurs is similar in the presence or absence of TPA and FCA. However, with TPA and FCA absent, this compressed pulse is associated with an output pulse peak power of 15 W, which is considerably higher than when TPA and FCA is present. From Figure 6(b), it may be observed that as the input peak power of the pulses is increased beyond 7.2 W, the compressed pulse starts to broaden from its narrowest point and starts to develop pulse pedestals with increasing amplitude. At these power levels, the nonlinear phase acquisition continues to be efficient in the absence of TPA and FCA, resulting in the Bragg soliton evolving into a high-order soliton which splits over the length of the grating. For optimal temporal compression at these power levels, the length of the grating should be shorter to capture the regime of initial pulse narrowing where significant enhancement in the pulse peak power occurs, prior to the splitting of the high-order Bragg soliton.

A similar observation may be made when simulating the temporal pulse profile as a function of input peak power for configuration 2, using the conditions used for the experiments in Figure 5 and varying the input peak power from 0.001 W to 15 W. It may be observed from Figure 6(c) that the pulse gradually narrows when the input peak power is increased. A compressed pulse width of 1.1 ps corresponds to an input peak power of 11 W and output pulse peak power of 4.0 W. In the ideal scenario where the system is lossless and the compressed pulses are transform limited, the peak power increase should be proportional to the compression factor, $CF=\frac{Input\;Pulse\;FWHM}{Output\;Pulse\;FWHM}$ . In this case, nonlinear loss effects result in significant attenuation in the peak power of the pulses.

Figure 6(d) simulates the temporal pulse profile as a function of input peak power for configuration 2, using the conditions used for the experiments in Figure 5, but in the absence of TPA and FCA. In this case, the compressed pulses have a narrowest pulse width of 0.8 ps at an input peak power of 15 W, which is a compressed pulse width similar to the case with TPA and FCA. However, this is associated with a maximum peak power of 80 W, which is significantly larger than when TPA and FCA is present. The temporal compression dynamics for configurations 1 and 2 implemented on silicon, are efficient from the standpoint of narrowing the pulse width. However, simulations for both configurations confirm that the peak power of the compressed pulses suffers from significant impairment from TPA and FCA.

In this paper, we have explored the feasibility of implementing temporal compression systems based on high-resolution gratings using an 8-inch CMOS-foundry process. The resolution limitations intrinsic to the manufacturing process dictated the device design. The silicon-on-insulator based devices were used to demonstrate two types of nonlinear optical compression phenomena, namely Bragg soliton-effect compression and compression through decoupled self-phase modulation in a waveguide and dispersive temporal synchronization in a grating. The devices were experimentally shown to enable up to 3.6× compression of 1.4 ps optical pulses to a pulse width of 0.39 ps. It is further elucidated that resolution limitations in wafer-scale CMOS-foundry manufactured devices could be overcome through device design modifications. While silicon was used in this work thus resulting in reduced power efficiency as a result of TPA and FCA, reasonably good compression factors could be achieved. Today, there are several foundries providing wafer-scale manufacturing on high nonlinear figure of merit platforms such as silicon nitride and aluminium nitride [32], [33], [34], [35]. Silicon nitride and aluminum nitride possess similar nonlinear refractive indices of 2.4 × 10^−15 ^cm^2^/W [36], [37] and 2.4 × 10^−15 ^cm^2^/W [38] and no TPA and FCA at telecommunications wavelengths. Both materials are widely used for nonlinear optics given the availability of low loss devices. Their availability has greatly accelerated advancements in wide-scale manufacturing of photonic devices. One limitation is the low nonlinear refractive index associated with these platforms which necessitates very high powers or extremely long interaction lengths in non-resonant, waveguide-based nonlinear effects. In the future, foundry availability of high nonlinear figure of merit CMOS platforms with Kerr nonlinearities on par with or better than silicon [18], [39] could provide a useful alternative for the design and implementation of efficient, low power nonlinear devices to be implemented in wafer-scale foundry processing.
